# Evaluating cognitive profiles of patients undergoing clinical amyloid-PET imaging

**DOI:** 10.1093/braincomms/fcab035

**Published:** 2021-03-12

**Authors:** Flavia Loreto, Stephen Gunning, Mara Golemme, Hilary Watt, Neva Patel, Zarni Win, Christopher Carswell, Richard J Perry, Paresh A Malhotra

**Affiliations:** Department of Brain Sciences, Faculty of Medicine, Imperial College London, London W6 8RP, UK; Department of Neuropsychology, Imperial College Healthcare NHS Trust, London W6 8RF, UK; Department of Neurology, Imperial College Healthcare NHS Trust, London W6 8RF, UK; Department of Primary Care and Public Health, Faculty of Medicine, Imperial College London, London W6 8RP, UK; Department of Nuclear Medicine, Imperial College Healthcare NHS Trust, London W6 8RF, UK; Department of Nuclear Medicine, Imperial College Healthcare NHS Trust, London W6 8RF, UK; Department of Neurology, Imperial College Healthcare NHS Trust, London W6 8RF, UK; Department of Brain Sciences, Faculty of Medicine, Imperial College London, London W6 8RP, UK; Department of Neurology, Imperial College Healthcare NHS Trust, London W6 8RF, UK; Department of Brain Sciences, Faculty of Medicine, Imperial College London, London W6 8RP, UK; Department of Neurology, Imperial College Healthcare NHS Trust, London W6 8RF, UK; UK Dementia Research Institute Care Research and Technology Centre, Imperial College London and the University of Surrey, London W12 0NN, UK

**Keywords:** Alzheimer’s disease, dementia, neuropsychology, amyloid PET imaging, cognitive testing

## Abstract

Episodic memory impairment and brain amyloid-beta are two of the main hallmarks of Alzheimer’s Disease. In the clinical setting, these are often evaluated through neuropsychological testing and amyloid PET imaging, respectively. The use of amyloid PET in clinical practice is only indicated in patients with substantial diagnostic uncertainty due to atypical clinical presentation, multiple comorbidities and/or early age of onset. The relationship between amyloid-beta and cognition has been previously investigated, but no study has examined how neuropsychological features relate to the presence of amyloid pathology in the clinical population that meets the appropriate use criteria for amyloid PET imaging. In this study, we evaluated a clinical cohort of patients (*n* = 107) who presented at the Imperial Memory Clinic and were referred for clinical amyloid PET and neuropsychological assessment as part of their diagnostic workup. We compared the cognitive performance of amyloid-positive patients (Aβ-pos, *n* = 47) with that of stable amyloid-negative (stableAβ-neg, *n* = 26) and progressive amyloid-negative (progAβ-neg, *n* = 34) patients. The amyloid-positive group performed significantly worse than both amyloid-negative groups in the visuospatial and working memory domains. Episodic memory performance, however, effectively differentiated the amyloid-positive group from the stable but not the progressive amyloid-negative group. On affective questionnaires, the stable amyloid-negative group reported significantly higher levels of depression than the amyloid-positive group. In our clinical cohort, visuospatial dysfunction and working memory impairment were better indicators of amyloid positivity than episodic memory dysfunction. These findings highlight the limited value of isolated cognitive scores in patients with atypical clinical presentation, comorbidities and/or early age of onset.

## Teaser

Loreto et al. assess the cognitive profiles, measured by standard neuropsychological testing, of memory clinic patients undergoing clinical amyloid PET imaging. In this study, visuospatial and working memory impairment, rather than episodic memory, best differentiated patients with Alzheimer’s disease from those with non-Alzheimer’s disease neurodegenerative and non-Alzheimer’s disease non-neurodegenerative conditions.

## Graphical Abstract

**Graphical Abstract:**
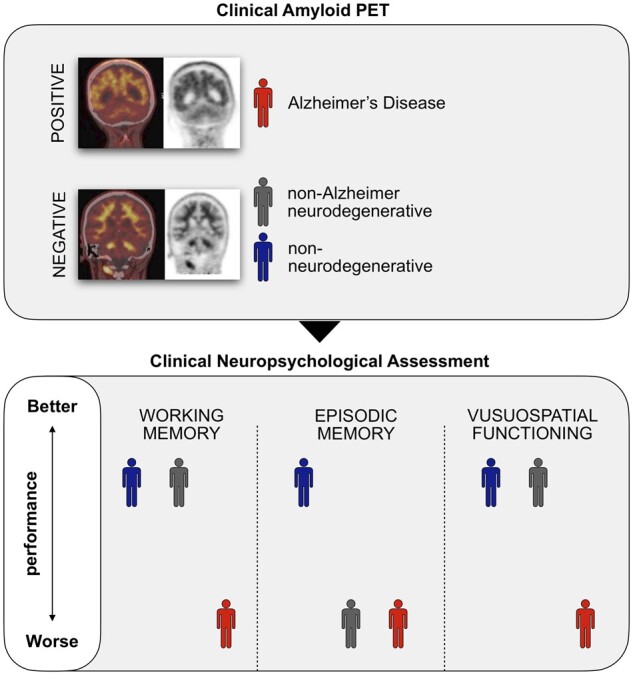

## Introduction

The onset of cognitive impairment in Alzheimer’s disease is typically associated with episodic memory decline, reflecting early medial temporal lobe (MTL) dysfunction.^1^ However, it is increasingly recognized that a substantial proportion of individuals present with atypical cognitive features,^2^ including predominant deficits in the visuospatial (Posterior Cortical Atrophy),^3^ language (Primary Progressive Aphasia)^4^ or behavioural^5^ domains. Further, young-onset Alzheimer’s disease, which is frequently associated with atypical presentations, is understood to have a higher incidence than previously thought.^6^

With the recognition of this clinical heterogeneity, brain biomarkers have acquired a more prominent role, as reflected in the revised diagnostic criteria for Alzheimer’s disease.^7^^,^^8^ In vivo detection of amyloid-beta (Aβ) deposition, one of the hallmarks of Alzheimer's disease, is enabled by cerebrospinal fluid analysis or by amyloid PET imaging (API). Three amyloid-binding PET ligands (Florbetapir, Flutemetamol and Florbetaben) are currently available for clinical use and have been shown to reliably detect increased amyloid plaque load in Alzheimer’s disease patients. Interpretation is binary, with a positive scan indicating increased burden of Aβ accumulation.^9^

API is used extensively in research but its use in clinical practice remains limited. Clinical implementation is guided by appropriate use criteria published by the Amyloid Imaging Taskforce, resulting from a joint initiative of the Society of Nuclear Medicine and Molecular Imaging and the Alzheimer’s association.^10^ These recommend using API in three categories of patients: (i) with persistent or progressive unexplained mild cognitive impairment (MCI), (ii) where Alzheimer’s disease is suspected but with atypical clinical course or aetiologically mixed presentation and (iii) with progressive dementia and atypically early age of onset.^10^ Thus, patients requiring clinical investigation through API constitute a definable clinical category, encompassing a spectrum of clinical phenotypes with atypical presentations and inconclusive findings on standard diagnostic tests.

A large proportion of individuals fulfilling appropriate use criteria for API are referred for neuropsychological assessment as part of the diagnostic process. For this reason, examining which neuropsychological characteristics relate to the presence of amyloid pathology in this group has important clinical implications. Some studies investigating amyloid status and cognitive performance have shown an association between memory decline and the presence of imaging^11^ and fluid^12^ biomarkers of Aβ in MCI. However, a recent meta-analysis, while confirming low memory scores as an early marker of amyloid positivity, concluded that these have limited validity as a screening measure for early forms of Alzheimer’s disease in individuals without dementia.^13^ Moreover, some studies have not found differences between amyloid-positive and amyloid-negative MCI patients on traditional measures of episodic memory.^14^^,^^15^

Most studies to-date have examined selected research cohorts, with cognitive and biomarker assessments being carried out specifically for research purposes. However, to understand the relationship between impaired cognition and amyloid positivity in clinical practice, it is necessary to examine patients fulfilling appropriate use criteria for API. This clinical population includes younger patients with atypical presentations, who often have comorbidities that further contribute to interindividual variability.^16^ Significant diagnostic delays and misdiagnosis frequently occur in this patient group,^17^ affecting the optimal clinical care and management of these individuals. By looking at real-life clinical cohorts, it is possible to gain greater insight into what role standard neuropsychological measures might play in the diagnostic workup of these patients. In addition, this allows the evaluation of the clinical and cognitive profiles of individuals presenting in a tertiary care setting with suspected dementia and atypical clinical presentation.

At the Imperial College Healthcare NHS Trust Memory Clinic, over 400 patients have undergone clinical API since 2013 and many of these were also referred for neuropsychological assessment. In the current study, we examined the cognitive profiles of patients meeting appropriate use criteria for API. The objectives were to examine which cognitive measures differentiate patients with Alzheimer’s pathology from patients (i) without Alzheimer’s pathology and with evidence of clinical progression and (ii) without Alzheimer’s pathology and without evidence of clinical progression. A broader objective was to characterize the different cognitive profiles in our clinical cohort. To our knowledge, this is the first study to examine clinical API in relation to comprehensive neuropsychological assessment in patients meeting the appropriate use criteria.

## Materials and methods

### Subjects

The present study included 113 patients, out of a total of 396 patients scanned between December 2013 and June 2019 at Imperial. Patients were eligible if the following conditions were met: API was recommended in line with appropriate use criteria; they received clinical follow-up at our clinic; they had at least 1 formal standalone clinical neuropsychological assessment evaluating ≥4 cognitive domains and conducted within 18 months of API. When patients received more than one neuropsychological assessment, the one temporally closest to API was taken into account. In line with the appropriate use criteria, all patients had objective cognitive impairment on initial cognitive screening and determination of amyloid status was expected to impact upon diagnostic confidence and patient management.^10^

In addition to the above examinations, all patients had a standard diagnostic workup, including medical history, neurological examination, structural imaging and a variable range of additional investigations (e.g. FDG–PET, cerebrospinal fluid and EEG). Clinical information was retrospectively collected through review of electronic and physical case records. Where necessary, additional information was gathered through individual case review with the responsible clinician.

**Figure 1:**
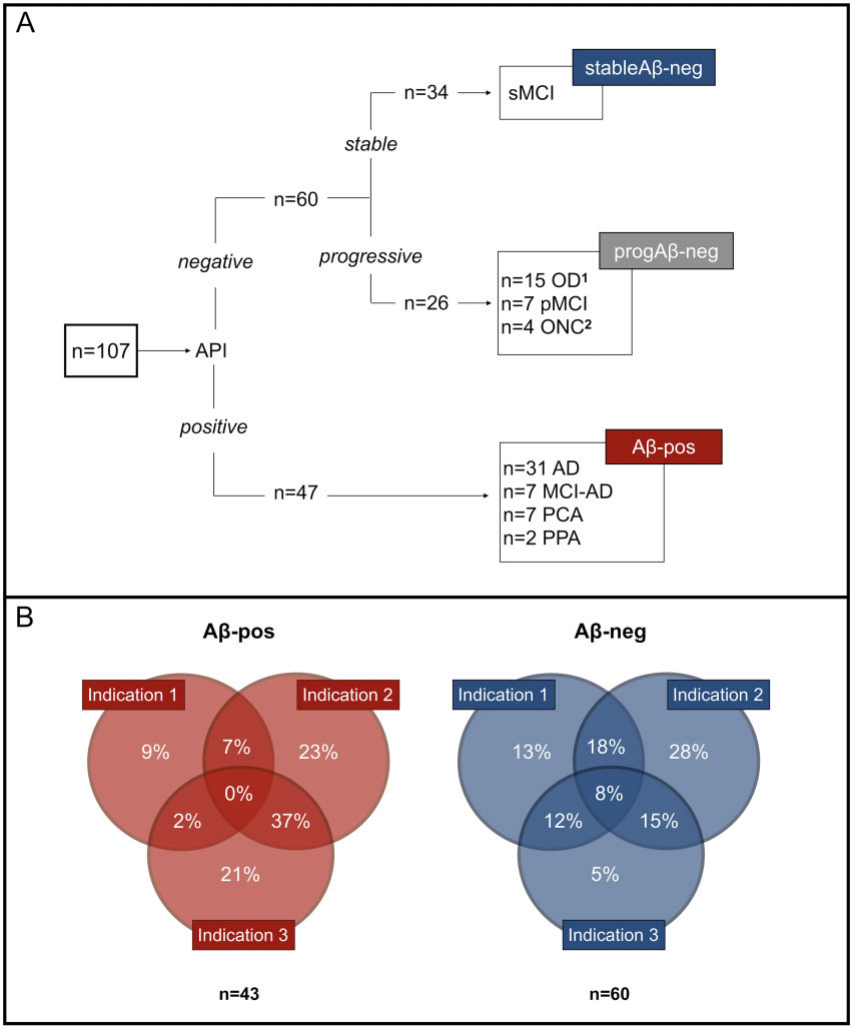
**Group definition flowchart and Appropriate Use Criteria classification. **(**A**) This figure illustrates the group characterization and sample sizes. The amyloid-negative (Aβ-neg) group was divided into progressive (progAβ-neg) and stable (stableAβ-neg) based on the presence or absence of clinical progression and/or concomitant neurological condition. Further details on the progAβ-neg group are as follows. ^1^OD (*n* = 15): FTD (*n* = 6), PSP (*n* = 1), CBS (*n* = 1), primary progressive MS (*n* = 1), progressive dementia with DLB features (*n* = 3), dementia secondary to post-radiation encephalopathy (*n* = 1) and progressive dementia with Parkinsonian features (*n* = 2). ^2^ONC (*n* = 4): NPH (*n* = 1), MS (*n* = 1), intracerebral metastases (*n* = 1) and stroke (*n* = 1). AD = Alzheimer's disease; MCI-AD = mild cognitive impairment due to Alzheimer's disease; PCA = posterior cortical atrophy; PPA = primary progressive aphasia; sMCI = stable mild cognitive impairment; OD = other dementia; pMCI = progressive mild cognitive impairment; ONC = other neurological condition; FTD = frontotemporal dementia; PSP = progressive supranuclear palsy; CBS = corticobasal syndrome; MS = multiple sclerosis; DLB = dementia with Lewy bodies; NPH = normal pressure hydrocephalus. (**B**) Reasons for referral to Amyloid-PET imaging in the amyloid-positive and amyloid-negative groups. Appropriate use criteria are defined as per to Johnson et al.^10^*Indication* 1: Patients with persistent or progressive unexplained MCI. *Indication* 2: Patients with suspected Alzheimer's Disease but with atypical clinical course or aetiologically mixed presentation. *Indication* 3: Patients with progressive dementia and atypically early age of onset (<65 age years)

Of the 113 patients, 47 were amyloid-positive (Aβ-pos) and 66 amyloid-negative (Aβ-neg). Six patients had to be excluded due to the unavailability of recent medical records. A total of 107 (47 Aβ-pos and 60 Aβ-neg) individuals were included in our initial analysis. Aβ-neg patients were further divided into two sub-groups: ‘progressive’ and ‘stable’, with the former being based on clinical progression and/or the presence of a concomitant neurological condition known to affect cognitive function (Fig. 1A). Clinical progression was defined as objective decline in patient’s cognition and/or activities of daily living which was significant enough to be noted by the clinician at follow-up in the Imperial Memory Clinic. Characterization of the amyloid-negative group was retrospectively performed by the study team through the systematic review of clinical correspondence; unclear cases were discussed with clinicians. Information gathered through the neuropsychological assessments analysed in this study were not used to define group membership in order to avoid circularity.

The ‘progressive amyloid-negative’ (progAβ-neg) group (*n* = 26) included patients with progressive MCI^18^ (*n* = 7), other dementia (*n* = 15) and other neurological conditions (ONC, *n* = 4) (further details in Fig. 1A). Due to the risks linked with the communication of clinical diagnosis of MCI,^19^ this was not always explicitly reported in the case record. In these cases, we retrospectively defined as MCI those patients whose clinical characteristics met Petersen and colleagues’ diagnostic criteria.^18^ Patients were classified as ‘stable amyloid-negative’ (stableAβ-neg) if there was no clinical evidence of progressive cognitive impairment and preserved ability to carry out activities of daily living at follow-up (*n* = 34). The underlying cause of cognitive impairment in this group remained unclear or was attributed to psychological factors, including stress, anxiety and depression. Patients with positive API (Aβ-pos) were given a clinical diagnosis of Alzheimer’s disease (*n* = 31), MCI due to Alzheimer’s disease (*n* = 7), primary progressive aphasia (*n* = 2) or posterior cortical atrophy (*n* = 7) secondary to Alzheimer’s pathology. It is important to note that, although Alzheimer’s pathology was felt to be the primary cause of cognitive deterioration, in some cases co-morbidities (e.g. multiple sclerosis and small vessel disease) could contribute to the observed impairment.

### Neuropsychological assessment

Formal neuropsychological assessment was carried out after clinical referral by a Dementia specialist, and conducted by a qualified clinical psychologist or neuropsychologist on a separate day from medical evaluation. Due to the neuropsychological assessment being conducted solely for clinical purposes, test batteries varied across patients. Hence, we limited our analysis to measures that were administered to at least 65% of the sample.

Neuropsychological tests and cognitive domains are detailed in Table 1. Both raw scores and Scaled Scores (SS) were available. SS are age-adjusted scores based on published norms and frequently used in the clinical setting to evaluate the clinical significance of patients’ performance. We analysed raw scores to evaluate statistical differences in groups’ cognitive performance and SS to characterize patients’ cognitive profiles within the clinical context. In addition to cognitive tests, affective symptoms were evaluated with the Hospital Anxiety and Depression Scale.^24^

**Table 1:** Cognitive domains and tests analysed in this study

Domain	Cognitive test
Premorbid functioning	Predicted full scale IQ
Confrontation naming	Graded naming test^20^
Short-term memory	Digit span forward^21^
Working memory	Digit span backward^21^
	Digit span sequencing^21^
Verbal memory and learning	Logical memory immediate recall^21^
	Logical memory delayed recall^21^
Verbal fluency	Verbal fluency letters^22^
	Verbal fluency categories^22^
Executive functioning	Verbal fluency switching accuracy^22^
Visuospatial functioning	Block design^23^
Abstract reasoning	Similarities^23^

#### Amyloid PET: referral and neuroimaging procedure

The decision to perform API was made by consensus by the multidisciplinary team,^16^ as per Johnson and colleagues.^10^ All cases met one or more appropriate use criteria. For 96% (*n* = 103) of referrals, we were able to specify which indications were met (Fig. 1B). Patients were scanned at Imperial College Healthcare NHS Trust (London, UK). The PET ligand changed from (18) F-florbetapir to (18) F-florbetaben in December 2017, following cessation of UK (18) F-florbetapir manufacture. For both tracers, a Siemens Biograph 64 PET/CT scanner was used; for (18) F-florbetapir, a 20-min acquisition of the brain was obtained following a 40-min interval post-injection of an intravenous bolus of 370 mBq; for (18) F-florbetaben, a 30-min brain acquisition was obtained following a 90-min interval post-injection of an intravenous bolus of 360 mBq. All images were visually read as ‘amyloid-positive’ or ‘amyloid-negative’ by an experienced nuclear medicine radiologist using greyscale images and the cerebellum as the reference region. Equivocal cases were independently read by two nuclear medicine radiologists and by a third reader when there was disagreement. The majority of scans (79%) included in this study were ‘type A’, of typical appearance.^9^

### Statistical analysis

Analyses were conducted using SPSS (version 26.0). Missing data were not estimated. The proportion of data available for each cognitive measure is shown in Table 2. Continuous variables were tested for normality using the Shapiro–Wilk test. Normality assumption was met by demographic and cognitive variables but violated by the affective symptom variables, which were analysed with non-parametric tests. Differences in demographics and general characteristics across the three groups (amyloid positive, *Aβ-pos*; stable amyloid-negative, *stableAβ-neg*; progressive amyloid-negative, *progAβ-neg*) were examined by mean of analysis of variance and Pearson’s *χ*^2^ test. Analyses of covariance models (with age, sex and premorbid IQ as covariates and with group as fixed factor) examined differences in performance for each cognitive measure. Significant *P*-values were adjusted through Bonferroni correction for comparison across the 11 cognitive measures considered (adjusted *P *=* P*/11). Bonferroni-adjusted *posthoc* comparisons (comparing two groups at a time) were performed when *P* < 0.05 for group. To evaluate whether age moderated the association between amyloid and cognitive performance, we conducted a factorial analysis of covariance with clinical group (Aβ-pos; stableAβ-neg and progAβ-neg), age group (< 65 or >65) and their interaction as the fixed factors, with sex and premorbid IQ as covariates. The partial eta squared (*η*^2^) is reported as an index of effect size. We further characterized the cohort’s cognitive profiles by examining the frequency and the pattern of impairment across all cognitive domains in the three groups. Impairment was defined as a SS less than or equal to 4, corresponding to 2 SDs below the mean. We then tested whether the frequency of impairment differed significantly between the Aβ-pos and progAβ-neg groups using Pearson’s *χ*^2^ test. Differences in anxiety and depressive symptoms were analysed using the Kruskal–Wallis test (using the Mann–Whitney U test to find *P*-values for each pairwise comparison of groups when the overall group comparison resulted in *P* < 0.05). We used Pearson’s *χ*^2^ test to compare the frequency of clinical levels of depression and anxiety (Hospital Anxiety and Depression Scale ≥ 8) between the three groups.

**Table 2:** Unadjusted mean raw scores obtained by the three groups on analysed cognitive measures

Cognitive measure	Aβ-pos (*n* = 44)		stableAβ-neg (*n* = 30)		progAβ-neg (*n* = 25)	
	*N*% valid	Mean ± SD	*N*% valid	Mean ± SD	*N*% valid	Mean ± SD
pFSIQ	93.6	101.27 ± 12.3	88.2	101.93 ± 13.45	96.2	100.96 ± 11.95
Graded naming test	74.5	14.43 ± 6.47	67.6	18.09 ± 6.47	76.9	16.2 ± 6.91
DS forward	91.5	8.58 ± 2.58	100.0	8.29 ± 2.82	92.3	8.17 ± 1.83
DS backward	91.5	5.88 ± 2.56	100.0	6.97 ± 2.48	92.3	6.21 ± 2.19
DS sequencing	87.2	4.39 ± 2.96^b^^,^^c^	91.2	6.48 ± 2.68^a^	92.3	6.00 ± 2.54^a^
LM immediate recall	80.9	16.03 ± 11.96	79.4	24 ± 8.56	84.6	19.09 ± 13.3
LM delayed recall	76.6	7.22 ± 8.04^b^	79.4	15 ± 9.48^a^	84.6	9.09 ± 6.26
VF letters	91.5	30.14 ± 15.61	79.4	30.7 ± 13.44	88.5	23.22 ± 12.28
VF category	95.7	23.38 ± 8.51	97.1	27.03 ± 9.39	100.0	21.42 ± 10.25
VF switching accuracy	78.7	6.24 ± 4.0	67.6	9.48 ± 3.5	80.8	7.19 ± 4.09
Block design	91.5	20.4 ± 13.63^b^^,^^c^	88.2	30.43 ± 10.69^a^	92.3	27.79 ± 9.81^a^
Similarities	76.6	19.67 ± 6.49	61.8	20.62 ± 6.88	100	19.1 ± 7.67

Aβ-pos = amyloid-positive; stableAβ-neg = stable amyloid-negative; progAβ-neg = progressive amyloid-negative; pFSIQ = predicted full-scale IQ; DS = Digit span; LM = Logical memory and VF = Verbal fluency.

Bonferroni adjusted *P* = *P*/11.

a Significantly different from Aβ-pos.

b Significantly different from stableAβ-neg.

c Significantly different from progAβ-neg.

#### Standard protocol approvals, registrations and patient consents

Access to anonymized clinical data for this study received approval by UK Research Ethics Committeee.

#### Data availability

Data not provided in the article are available upon request.

## Results

### Demographic data

As shown in Table 3, the three groups did not differ in age and premorbid functioning at the time of cognitive assessment (*P* = 0.76 and 0.96, respectively). Mean age did not differ between the amyloid-positive (age years = 66.6 ± 8.8) and the amyloid-negative (age years = 67.4 ± 9.7) patients (*P* = 0.65). There was a significant association between group and sex, with a lower percentage of females in the stableAβ-neg group compared to the other two groups (*χ*^2^_(2)_ = 8.26, *P* = 0.016). The total length of clinical follow-up, computed as the number of days between the first and the last visit to our Centre, was available for 96% (*n* = 103) of the sample: the medians were 957 days, 822.5 days and 882 days for the Aβ-pos, stableAβ-neg and progAβ-neg groups, respectively (Kruskal–Wallis *H*_(2)_ = 1.185, *P* = 0.55).

**Table 3:** Demographic and general characteristics of the study sample

		**Aβ-pos** **(*n* = 47)**	**stableAβ-neg** **(*n* = 34)**	**progAβ-neg** **(*n* = 26)**
Age at cognitive assessment, years	*Mean ± SD*	66.57 ± 8.84	68.03 ± 10.48	66.58 ± 8.71
	*Median*	66	67	69
	*Range*	42–86	45–85	44–79
Gender, *female*	%	61.70%	29.4%	50%
English first language	%	83%	70.6%	80.8%
Interval API/Cognitive assessment, days	* Mean ± SD*	57.49 ± 180.02	57.76 ± 227.58	87.85 ± 203.26
	* Median*	72	40	64
	*Range*	–530/+527	–474/+428	–250/+507
NPS preceding API	*Frequency*(%)	35(74%)	18(53%)	19(73%)
Follow-up length, *days*	*Median*	957	822.5	882
N. Cognitive tests administered	*Mean± SD*	14.6 ± 3.64	14.82 ± 2.4	15.27 ± 2.31
	*Range*	6/18	10/18	9/18
N. Cognitive domains tested	*Mean ± SD*	7.62 ± 1.51	7.82 ± 1.11	8.12 ± 1.18
	*Range*	4/9	6/9	5/9

Aβ-pos = amyloid-positive; stableAβ-neg = stable amyloid-negative; progAβ-neg = progressive amyloid-negative; Aβ-PET = Amyloid PET; NPS = neuropsychological assessment.

There was a comparable proportion of patients in each group whose first language was not English (*P* = 0.39).

### Neuropsychological assessment

Neuropsychological assessments preceded API in 67.3% cases and followed API in 32.7% cases, with no significant difference across the three groups as to when examinations were carried out in relation to each other (*χ*^2^_(2)_ = 4.68, *P* = 0.097) (Table 3). The interval between cognitive assessment and API did not differ significantly between groups. All three groups were administered a comparable number of cognitive tests, and there was no difference in the mean number of cognitive domains assessed.

### Cognitive measures

**Figure 2:**
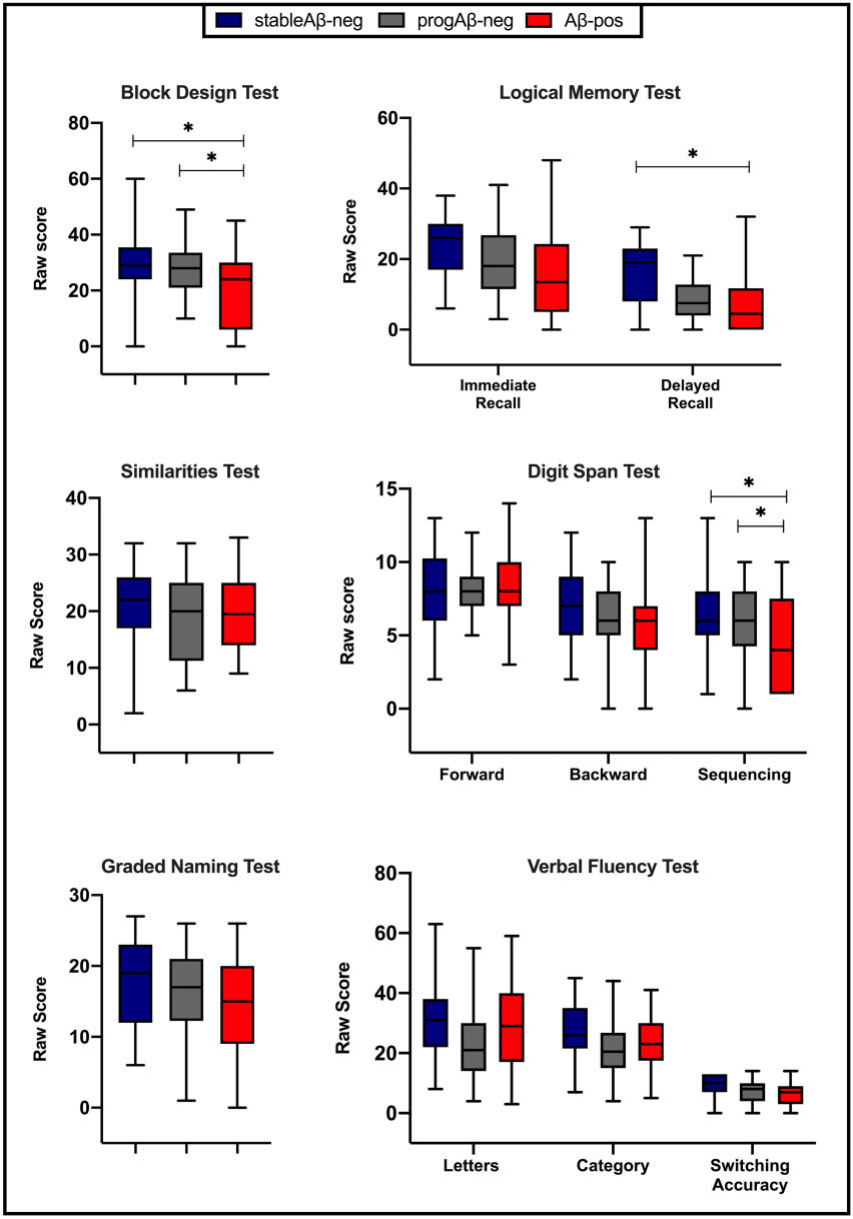
**Comparison of cognitive performance by group.** Unadjusted mean raw scores of cognitive measures. *Adjusted *P* < 0.05.

The predicted full-scale IQ score was not available for eight patients. Therefore, a total of 99 patients were assessed for group differences in cognitive performance. After controlling for age, sex and premorbid functioning and adjusting for multiple comparisons, a main effect of group was found for three measures (Fig. 2): visuospatial functioning (Block Design, *F*_(2,86)_ = 7.05; *P* = 0.01; *partial η*^2^ = 0.14), verbal episodic memory (Logical Memory Delayed Recall, *F*_(2,77)_ = 5.94; *P* = 0.04; *partial η*^2^ = 0.13) and working memory (Digit Span Sequencing, *F*_(2,83)_ = 7.77; *P* = 0.01; *partial η*^2^ = 0.15). *Posthoc* comparisons revealed that the Aβ-pos group performed worse than both the stableAβ-neg and progAβ-neg groups on the Block Design Task (*P* = 0.002 and *P* = 0.03, respectively) and on the Digit Span Sequencing (*P* = 0.002 and *P* = 0.02, respectively) tasks, with no difference between the two negative groups (Table 2). A different pattern was observed for the verbal episodic memory measure: the Aβ-pos group’s mean score was lower than the stableAβ-neg group (*P* = 0.003), but comparable to the progAβ-neg group; the difference between the progAβ-neg group and the stableAβ-neg group was not significant (*P* = 0.07). The covariate predicted full-scale IQ had an effect on all cognitive measures (*P* consistently < 0.05), showing a positive association between premorbid functioning and cognitive score (i.e. the higher the premorbid IQ, the better the cognitive performance). The factorial analysis of covariance revealed no significant interactions between age and group on any cognitive measure.

### Characterization of cognitive impairment

**Figure 3:**
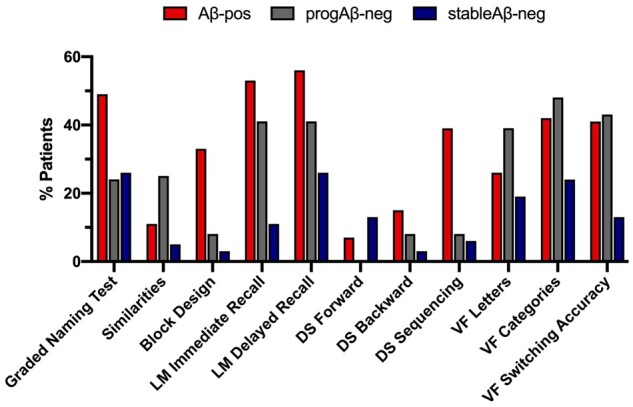
**Frequency of impairment across cognitive domains by group.** Proportion of patients impaired (i.e., SS ≤ 4) in each cognitive measure across groups. LM = logical memory; DS = digit span; VF = verbal fluency.

Learning and episodic memory were the most frequently impaired measures in the amyloid-positive group (Fig. 3). Executive functioning and semantic retrieval were the most impaired in the progAβ-neg group, followed by learning and episodic memory. Overall, the stableAβ-neg showed the lowest proportion of impairment, with the most frequently impaired measures reaching approximately 20%. When comparing the frequency of impairment between the Aβ-pos and the progAβ-neg groups, we found that the two were comparable for most cognitive measures including learning (*χ*^2^_(1)_ = 0.767, *P* = 0.38) and episodic memory (*χ*^2^_(2)_ = 1.17, *P* = 0.28). In line with quantitative results, the Aβ-pos group was more frequently impaired than the progAβ-neg group in visuospatial functioning (*χ*^2^_(1)_ = 5.2, *P* = 0.023) and working memory (*χ*^2^_(1)_ = 7.54, *P* = 0.006).

**Figure 4:**
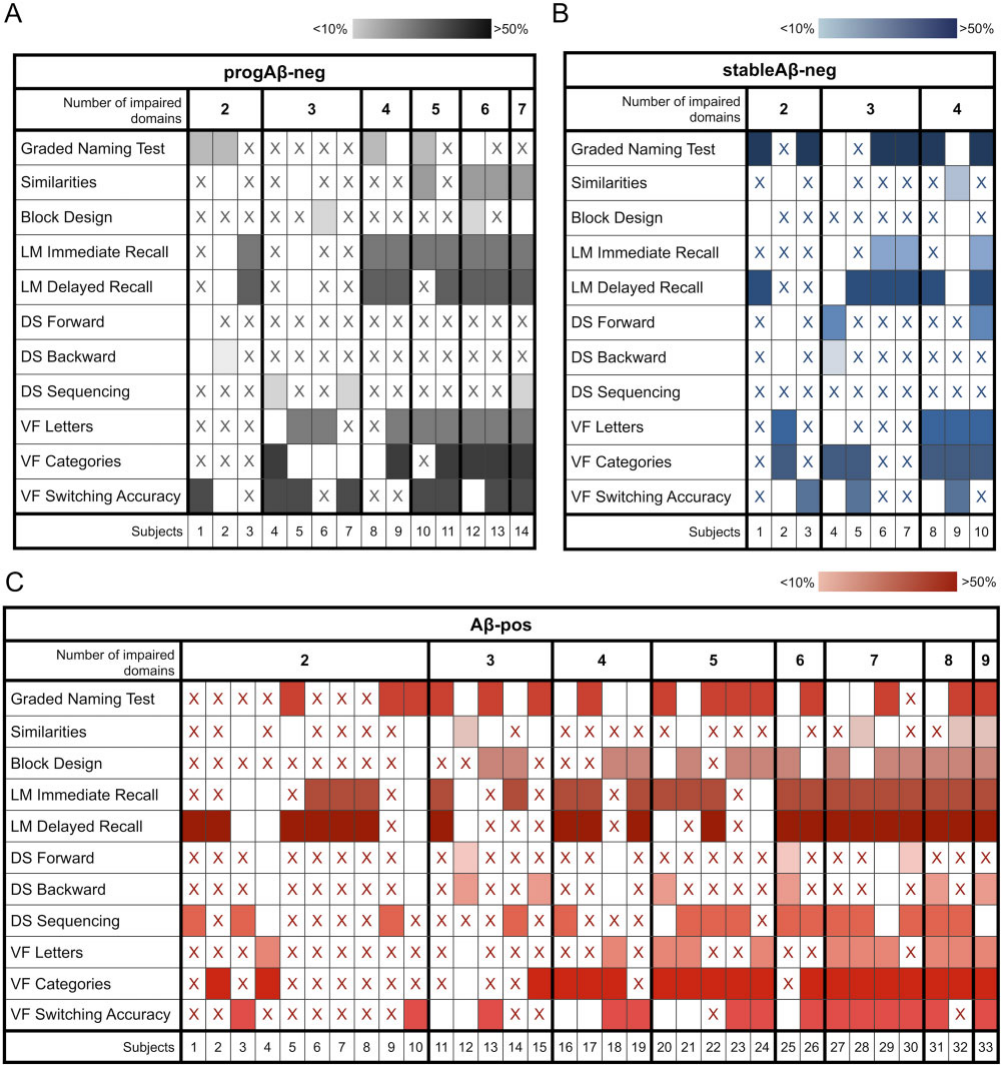
**Patterns of cognitive impairment.** Patterns of cognitive impairment in the progAβ-neg in **A**, stableAβ-neg in **B** and Aβ-pos in **C** groups. Note that only patients with impairment in at least two cognitive domains are represented here. The ‘*x*’ indicates that cognitive performance on that test was not impaired; empty cells indicate that the measure was not administered and coloured boxes indicate that performance on that test was impaired. The darker the colour, the higher the proportion of impaired patients in that measure in each group.

We determined how many patients in each group had cognitive scores below 2 SDs for up to the 0%, 25%, 50%, 75% and 100% of the cognitive domains assessed. Approximately one-fifth of the Aβ-pos and of the progAβ-neg had no scores below this cut-off in any of the domains assessed (Table 4). Figure 4 shows cognitive performance patterns in patients with impairment in two or more cognitive domains.

**Table 4:** Proportion of impaired domains

% impaired domains	**Aβ-pos** **(*n* = 44)**	**stableAβ-neg** **(*n* = 30)**	**progAβ-neg** **(*n* = 25)**
	*n* (%)	*n* (%)	*n* (%)
**0**	10 (21%)	16 (47%)	6 (23%)
**1–25**	12 (26%)	9 (26%)	7 (27%)
**26–50**	12 (26%)	6 (18%)	10 (38%)
**51–75**	9 (19%)	3 (9%)	3 (12%)
**76–100**	4 (9%)	0	0

Aβ-pos = amyloid-positive; stableAβ-neg = stable amyloid-negative and progAβ-neg = progressive amyloid-negative.

Number of patients in each group with impairment (i.e. SS ≤ 4) in 0, 1–25%, 26–50%, 51–75% or 76–100% of the domains assessed.

### Affective symptoms

Hospital Anxiety and Depression Scale scores were available for 79% (*n* = 85: 36 Aβ-pos, 29 stableAβ-neg, 20 progAβ-neg) of patients. A substantial proportion of patients scored above the clinical cut-off for anxiety and depression, with comparable frequency across all groups (*χ*^2^_(2)_=1.142, *P* > 0.05 and *χ*^2^_(2)_= 2.322, *P* > 0.05, respectively). Notably, in all three groups a significant proportion of patients reported a score above the clinical cut-off for both anxiety (Aβ-pos 65.96%; progAβ-neg 53.85%; stableAβ-neg 64.7%) and depression (Aβ-pos 36.17%; progAβ-neg 46.15%; stableAβ-neg 52.94%). Analysis of mean scores revealed an effect of group on depression (*H*_(2)_ = 9.79, *P* = 0.007) but not on anxiety. *Post hoc* analyses showed that this effect was due to the stableAβ-neg group reporting significantly higher levels of depression compared to the Aβ-pos group (mean ± SD: StableAβ-neg 7.62 ± 4.23; Aβ-pos 4.47 ± 3.53; adjusted *P* = 0.006).

## Discussion

In this study, we assessed the cognitive profiles of patients undergoing API in line with appropriate use criteria.^10^ In 99 patients who had clinical API and formal neuropsychological assessment, we found that both the stable and the progressive amyloid-negative groups did not differ from the amyloid-positive group in most measures. Episodic memory, the most affected cognitive domain in typical Alzheimer’s disease, was also the most frequently affected domain in our amyloid-positive group. However, the frequency of episodic memory impairment was at a comparable level in the progressive amyloid-negative group. The amyloid-positive group differed from both amyloid-negative groups on the Block Design Test, which probes visuospatial and constructional ability, and Digit Span Sequencing, a measure of working memory. Although there was a positive relationship between estimated premorbid functioning and cognitive performance, it should be noted that the reading test-based predicted full-scale IQ, whilst generally resistant to cognitive decline, can lead to underestimation of premorbid functioning in dementia, potentially explaining this observation.

The visuospatial dysfunction observed in the amyloid-positive group is consistent with evidence that impairment in this domain is an early, often overlooked, sign of Alzheimer’s disease^25^ with good diagnostic potential.^26^ Visuospatial deficits have been linked to parietal dysfunction, most often seen in young-onset and atypical forms of Alzheimer’s disease.^27^ The amyloid-positive group also showed working memory impairment in the sequencing but not in the forward or backward subtests of the Digit Span Test, suggesting specificity for Alzheimer’s pathology in this patient group. The sequencing component of this test probes the ability to hold and manipulate information, with a higher working memory load and a greater executive component.^28^ This finding is in keeping with work showing higher sensitivity of this measure to working memory impairment in Alzheimer’s disease^29^ and supports the use of this subtest in this group.

Notably, episodic memory was not significantly different in amyloid-positive and progressive amyloid-negative patients, with both groups demonstrating worse performance and more frequent impairment than the stable amyloid-negative group. Episodic memory has been traditionally considered an early marker of Alzheimer’s disease in typical presentations with prominent MTL involvement,^1^ but our findings would discourage using impairment on episodic memory tasks to infer the presence of Alzheimer’s pathology in patients fulfilling API appropriate use criteria. This is in keeping with the observation that amnestic deficits seem to have relatively low specificity for Alzheimer’s pathology,^30^ being observed in a number of other brain diseases.^31^^,^^32^ This is particularly evident in the cohort with atypical features and/or younger onset described here.^10^

The recent IDEAS Study evaluated a large cohort of patients fulfilling the appropriate use criteria and showed that API led to substantial changes in the diagnosis and management of this clinical population.^33^ By examining neuropsychological assessment in combination with API within the clinical setting, our results provide direct insight into the role neuropsychological testing might play in patients eligible for clinical API. Spallazzi and colleagues found that API had minimal impact on diagnostic change in a group of memory clinic patients, and suggested that this was attributable to the inclusion of a comprehensive neuropsychological assessment in their diagnostic workup.^34^ It has also been proposed that neuropsychological testing alone can provide similar information to API in some groups.^35^ Our results suggest that this is not the case in this population.

Given that neuropsychology is more accessible than API, it is essential to understand how it can contribute most effectively to management. It is critical to note that test scores alone are of limited value and their contribution to diagnosis relies on expert interpretation. Results must be considered alongside a thorough history, collateral account, neurological examination, imaging and other investigations. In our experience neuropsychological testing is also particularly helpful in monitoring progression and helping to clarify the underlying aetiology in non-AD dementia.^36^ It plays a key role in the follow-up of amyloid-negative patients, where diagnostic uncertainty often persists following API. Although we did not assess longitudinal cognitive data here, 20% of the amyloid-negative group (versus 11% of the amyloid-positive group) received at least one follow-up cognitive assessment following API. In this group, repeated cognitive assessments over 6–12 months may establish whether impairment is progressive.

Depressive symptoms are often part of the clinical presentation of dementia.^37^ These can also be associated with cognitive underfunctioning that mimics a dementia profile in otherwise healthy elderly individuals,^38^ adding complexity to the identification of the underlying aetiology of impairment. Notably, the presence of depression and anxiety is often one of the exclusion criteria of prospective observational studies. In our study, about 30% of patients who received clinical API were amyloid-negative, with stable cognitive deficits and moderate levels of anxiety and depression. This suggests that psychological factors are likely to contribute to the cognitive impairment seen in a considerable proportion of patients undergoing clinical API. Therefore, in this group of patients, neuropsychological evaluation and amyloid PET may be synergistic in identifying a causative role for psychological factors and ruling out underlying Alzheimer’s disease.

In our cohort, the amyloid-positive group had the lowest levels of depression and the fewest cases scoring above the clinical cut-off for the depression subscale. Conversely, the symptoms of anxiety in the amyloid-positive and the progressive amyloid-negative groups were of larger magnitude and frequency than the stable amyloid-negative individuals. These findings highlight a possible link between symptomatic anxiety and neurodegeneration, but the relationship between dementia and neuropsychiatric symptoms is clearly complex and bidirectional^39^ and requires further investigation.

## Limitations

The main limitations of this study relate to its retrospective nature. Since the neuropsychological assessments were carried out for clinical purposes, tests were selected at clinical discretion leading to variability in the range of tests performed. We addressed this problem by selecting only those measures that were consistently administered to at least 65% of patients. However, it was not possible to include a measure of visual memory and a measure of cued or recognition memory. As impairment in the visual memory domain has been consistently linked to typical Alzheimer’s disease,^40^ further studies are needed to investigate whether these deficits extend to atypical presentations. Similarly, it would also be of importance to determine whether recognition and cued memory tests, which better control for the effect of encoding,^41^^,^^42^ have higher specificity for Alzheimer’s pathology in patients fulfilling the appropriate use criteria. In fact, patients meeting these criteria form a group that, by its very nature, differs from patients with typical clinical features. In light of this, caution should be taken in generalizing these findings to the wider clinical population of individuals with suspected Alzheimer’s disease.

## Conclusions

In an unselected memory clinic cohort presenting with atypical clinical features and/or early age of onset, we evaluated the role of available cognitive measures in differentiating amyloid-positive Alzheimer’s disease patients from amyloid-negative patients with a stable or progressive pattern of cognitive impairment. A measure of visuospatial functioning and a measure of working memory effectively differentiated the amyloid-positive group from both amyloid-negative groups. However, the remaining measures, including episodic memory, had limited value in differentiating between amyloid-positive and amyloid-negative patients with progressive cognitive impairment. Test scores should not be evaluated in isolation but in the context of a neuropsychological assessment that contributes to differential diagnosis alongside clinical assessment and investigations. By examining amyloid PET and neuropsychological assessment within the clinical context, this study adds to the existing literature on the diagnostic use of these examinations in patients with atypical presentations characterized by diagnostic uncertainty.

## Funding

The work is funded by the Alzheimer’s Society (grant no. P75464) and supported by the National Institute for Health Research (NIHR) Biomedical Research Centre at Imperial College London.

## Competing interests

Zarni Win previously participated in the Eli Lilly PET advisory board and was an amyloid PET read trainer. Christopher Carswell has taken part in an advisory panel for Roche pharmaceuticals. Richard Perry previously sat on an advisory board for Eli Lilly and received support from GE for research imaging from 2014 to 2018. Paresh Malhotra has given an educational talk at a meeting organized by GE. None of the authors currently has funding or support from any commercial organization involved in API.

## Abbreviations

A =: amyloid-beta
Aβ-pos =: amyloid-positive
AD =: Alzheimer’s disease
API =: amyloid PET imaging
CSF =: cerebrospinal fluid
HADS =: hospital anxiety and depression scale
MCI =: mild cognitive impairment
MTL =: medial temporal lobe
OD =: other dementia
ONC =: other neurological conditions
PCA =: posterior cortical atrophy
pFSIQ =: predicted Full-Scale IQ
pMCI =: progressive mild cognitive impairment
PPA =: primary progressive aphasia
progAβ-neg =: progressive amyloid-negative
sMCI =: stable mild cognitive impairment
SS =: scaled scores
stableAβ-neg =: stable amyloid-negative
